# Presence of Antibodies to SARS-CoV-2 in Domestic Cats in Istanbul, Turkey, Before and After COVID-19 Pandemic

**DOI:** 10.3389/fvets.2021.707368

**Published:** 2021-10-12

**Authors:** Aysun Yilmaz, Abdullah Kayar, Nuri Turan, Onur Iskefli, Alper Bayrakal, Gleyder Roman-Sosa, Erman Or, Hasan Emre Tali, Bekir Kocazeybek, Ridvan Karaali, Dashzeveg Bold, Jean-Remy Sadeyen, Deimante Lukosaityte, Pengxiang Chang, Munir Iqbal, Juergen A. Richt, Huseyin Yilmaz

**Affiliations:** ^1^Department of Virology, Veterinary Faculty, Istanbul University-Cerrahpasa, Istanbul, Turkey; ^2^Department of Internal Medicine, Veterinary Faculty, Istanbul University-Cerrahpasa, Istanbul, Turkey; ^3^Department of Diagnostic Medicine and Pathobiology, College of Veterinary Medicine, Kansas State University, Manhattan, KS, United States; ^4^Department of Medical Microbiology, Cerrahpasa Faculty of Medicine, Istanbul University-Cerrahpasa, Istanbul, Turkey; ^5^Department of Infectious Diseases and Clinical Microbiology, Cerrahpasa Faculty of Medicine, Istanbul University-Cerrahpasa, Istanbul, Turkey; ^6^Avian Influenza Group, The Pirbright Institute, Woking, United Kingdom

**Keywords:** SARS-CoV-2, spike, RBD, ELISA, cat, Turkey

## Abstract

Recent studies demonstrated that domestic cats can be naturally and experimentally infected with severe acute respiratory syndrome coronavirus-2 (SARS-CoV-2). This study was performed to investigate the presence of SARS-CoV-2-specific antibodies within the domestic cat population in Istanbul, Turkey, before the coronavirus disease 2019 (COVID-19) and during the COVID-19 pandemic. Overall, from 155 cat sera analyzed, 26.45% (41/155) tested positive in the spike protein-ELISA (S-ELISA), 28.38% (44/155) in the receptor-binding domain-ELISA (RBD-ELISA), and 21.9% (34/155) in both, the S- and RBD-ELISAs. Twenty-seven of those were also positive for the presence of antibodies to feline coronavirus (FCoV). Among the 34 SARS-CoV-2-positive sera, three of those were positive on serum neutralization assay. Six of the 30 cats before COVID-19 and 28 of the 125 cats during COVID-19 were found to be seropositive. About 20% of ELISA-positive cats exhibited mainly respiratory, gastrointestinal, and renal signs and skin lesions. Hematocrit, hemoglobin, white blood cells, lymphocyte, and platelet numbers were low in about 30% of ELISA-positive cats. The number of neutrophils and monocytes were above normal values in about 20% of ELISA-positive cats. The liver enzyme alanine aminotransferase levels were high in 23.5% ELISA-positive cats. In conclusion, this is the first report describing antibodies specific to SARS-CoV-2 antigens (S and RBD) in cats in Istanbul, Turkey, indicating the risk for domestic cats to contract SARS-CoV-2 from owners and/or household members with COVID-19. This study and others show that COVID-19-positive pet owners should limit their contact with companion animals and that pets with respiratory signs should be monitored for SARS-CoV-2 infections.

## Introduction

High numbers of severe respiratory infections in humans were first reported in December 2019 in Wuhan, China; the etiological agent was later characterized as severe acute respiratory syndrome coronavirus 2 (SARS-CoV-2) as an enveloped single-stranded RNA virus classified as betacoronavirus in the *Coronaviridae* family, and the disease was designated coronavirus disease 2019 (COVID-19) by the World Health Organization (WHO) (1–3). The virus spread rapidly worldwide and became a pandemic disease that has been causing very serious threats to public health and economies globally (4–6).

SARS-CoV-2 is a zoonotic agent and was most likely transmitted from bats to humans; however, it is not well-understood which animals are susceptible to and can spread COVID-19. Susceptible animals may not only serve as a reservoir for disease posing a risk to humans but also drive further virus changes (1, 7). It has been demonstrated that SARS-CoV-2 uses the angiotensin-converting enzyme 2 (ACE2) as its cellular receptor (1). The ACE2 of a cat shares a high amino acid sequence identity (85.2%) with human ACE2 (7). Feline ACE2 differs at only four out of a total of 20 residues compared with the human ACE2 conforming receptor binding pocket. However, these residues are the key determinants for virus-host range differentiating the susceptibility of a species to SARS-CoV-2 and can affect the efficiency of receptor-binding domain (RBD) binding to ACE2 (8).

Considering the host receptor-virus relationship, the presence of SARS-CoV-2 antigen or antibodies against the virus has been reported in a considerable number of animals including but not limited to cats, dogs, minks, tigers, and lions (8–12). Among these, cats received great attention because of close contact with humans. Recent studies of SARS-CoV-2 infections in cats demonstrated that cats can be naturally and experimentally infected with SARS-CoV-2 (9, 11–14). Cats can be infected by two different coronaviruses including types I and II coronaviruses responsible for feline infectious peritonitis (7, 15, 16) or SARS-CoV-2 (8). SARS-CoV-2 is genetically distinct from the feline coronaviruses which are classified to belong to the alphacoronavirus genus (2). In addition, clinical, virological, pathological, and tomography findings of these infections are different from each other (16).

The first suspected case of a cat with SARS-CoV-2 was reported in Hong Kong by the Hong Kong Agriculture, Fisheries and Conservation Department (AFCD) (17). Another case of SARS-CoV-2 in cats was reported in Belgium in March 2020 (18) and later in other countries like the USA (19), France (20, 21), Germany (13), Italy (22), and Spain (23, 24). Infected animals can become sick, but the clinical disease seems to be mild and self-limited (18, 25). This was confirmed by experimental infections, indicating that SARS-CoV-2 causes mild respiratory signs in some instances (11); however, most cases remain asymptomatic (26). Importantly, infected cats were demonstrated to be capable of transmitting SARS-CoV-2 to other cats in the experimental environment (12).

Cats are very important companion animals living in close proximity to humans, and ~50% of people keep companion animals in Europe and the USA and in some localities in Turkey (27) (Turkish Veterinary Association, personal communication). Therefore, it is important to determine whether domestic cats pose a potential risk to people. Generally, ELISA, virus neutralization, and Western blot tests are used to investigate the serological response of cats to SARS-CoV-2. We investigated the presence of SARS-CoV-2-specific antibodies within the cat population in Istanbul, Turkey. For this purpose, *in-house* indirect ELISA tests were established using the recombinant spike (S) and RBD proteins of SARS-CoV-2. Then, the sera collected from domestic cats were subjected to the in-house ELISA tests in order to determine the presence of antibodies specific for S and RBD proteins of SARS-CoV-2.

## Materials and Methods

### Study Population, Clinical Examination, and Sampling

This study was performed on cats admitted to the Veterinary Faculty of Istanbul University-Cerrahpasa, Turkey, for clinical examination. All cats were household cats with no access to the street. The study population consisted of two groups of cats, one group named “before COVID-19” and the second group “during COVID-19.” Samples from the before COVID-19 group were collected between January 2018 and January 2019. The samples from the during COVID-19 group were collected after December 2019, the first reported SARS-CoV-2 human infection case in China.

The before COVID-19 group consisted of a total of 30 cats, and the during COVID-19 group consisted of 125 cats, between 2 months and 15 years of age. All cats were clinically examined and the gender, breed, and age of the animal were recorded. On clinical examination, presence of fever, depression, and clinical signs related to organ system dysfunction were recorded: respiratory (cough, wheezing, dyspnea, and abnormal lung sounds); gastrointestinal (mouth lesions, anorexia, vomitus, diarrhea, weight loss, abdominal distension, and/or ascites); circulatory (lymphadenopathy, anemia, icterus); urinary (cystitis, renal insufficiency, urinary infections); ocular lesions (conjunctivitis, keratic precipitates, uveitis, hyphema, iridocyclitis, chorioretinitis); skin lesions; and central nervous system (epileptic seizures, ataxia) symptoms. Sera from all cats were collected by venipuncture.

### Development of ELISA for Detection of Antibodies to S and RBD Proteins of SARS-CoV-*2*

The SARS-CoV-2 S and RBD proteins were initially produced and characterized in Professor Munir Iqbal's laboratory (The Pirbright Institute, UK) and in Professor Juergen Richt's laboratory (Kansas State University, Manhattan, KS, USA). Adopting methods established by the two labs, we produced both proteins in our laboratory.

Two methods were used to produce SARS-CoV-2 S and RBD proteins as described below:

**Method 1:** This method was used to produce SARS-CoV-2 S protein, as described previously (28, 29). Briefly, the expression cassettes containing SARS-CoV-2 S nucleotide sequences of BetaCov/Wuhan/WH04/2020 (accession number EPI-ISL-406801) were retrieved from the Global Initiative on Sharing All Influenza Data (GISAID) database. Nucleotide sequences were codon optimized for expression in *Drosophila melanogaster* Schneider 2 (S2) cells. The N-terminus signal sequence of S protein was replaced with Drosophila immunoglobulin heavy chain binding protein (BiP) signal sequence, and the C-terminus were fused with T4 foldon sequence (GSGYIPEAPRDGQAYVRKDGEWVLLSTFL) and a C-tag sequence (EPEA) for affinity purification. The protein expression cassettes were commercially synthesized (GeneArt, ThermoFisher Scientific, Regensburg, Germany) and cloned into pExpres2.1 expression vector (ExpreS2ion Biotechnologies, Hørsholm, Denmark). The recombinant plasmids were transfected into *Drosophila* S2 cells using Calcium Phosphate Transfection Kit (Thermo Fisher Scientific, Paisley, Scotland, UK). Following antibiotic selection with Zeocin (InvivoGen, Toulouse, France), cells were propagated in *Drosophila* EX-CELL® 420 Serum-Free Medium (Merck Life Science) at 25°C. Recombinant S trimeric protein secreted in cell culture supernatants was purified using the CaptureSelect™ C-tag Affinity Matrix (Thermo Fisher Scientific) and eluted protein dialyzed into PBS overnight. The concentration of purified recombinant S protein was determined by Pierce™ BCA Protein Assay Kit (Thermo Fisher Scientific), and the purity was assessed by sodium dodecyl sulphate-polyacrylamide gel electrophoresis (SDS-PAGE).

**Method 2:** This method was used to produce RBD region of the S protein in mammalian cells (human embryonic kidney 293 cells-HEK 293) in Professor Juergen Richt's laboratory (Kansas State University, Manhattan, KS). Briefly, the nucleotide sequence encoding the RBD spike protein of the SARS-CoV-2 isolate Wuhan-Hu-1 (GenBank accession: MT380725.1) plus two strep tags on the 3′end was used and cloned into the mammalian expression vector pHL (Addgene, Cambridge, MA, USA). The RBD recombinant protein was produced in HEK 293 cells by transfections of these cells with purified DNA using QIAGEN Plasmid Midi Kit (Qiagen, Germantown, MD, USA). Transfected HEK 293 cells were cultured in DMEM (Fisher Scientific, Chicago, IL, USA). Supernatants from transfected cells were harvested on day 3 posttransfection and supernatant samples were centrifuged at 4,000×*g* for 20 min. Recombinant proteins with strep-tags were purified *via* affinity chromatography using Strep-Tactin® (IBA Lifesciences, Göttingen, Germany) under native conditions using StrepTacting Superflow Agarose (Millipore Sigma, MA, USA) and expression of the recombinant protein was confirmed by Western blotting.

### Standardization of ELISA for Estimation of S and RBD Antibody Levels in Serum Samples Collected From Cats

In house, ELISA methods were standardized to analyze the sera of cats for the presence of antibodies to SARS-CoV-2 by using the purified recombinant S and RBD proteins of SARS-CoV-2 as the target antigen. Indirect ELISA protocols were standardized using checkerboard titration protocol, as described previously (30). For this, two-fold dilutions of the S and RBD proteins were prepared at a range of 250–4,000 ng/ml in PBS (Sigma) on 96-well ELISA plates (MaxiSorb flat bottom). The negative control sera were from the cats taken before the COVID-19 epidemic having low OD values and also being negative for the FCoV antibodies and in-house FCoV PCR. *Human sera which tested positive or negative for SARS-CoV-2 antibodies by a commercial ELISA (Roche, Basel, Switzerland) and human swab samples were positive for SARS-CoV-2 RNA by a real-time RT-PCR* (IDEXX, Westbrook, ME, USA) were included in the standardization of ELISA.

The SARS-CoV-2-positive and SARS-CoV-2-negative human sera were diluted two-fold (from 1:50 to 1:1,600), and HRP-conjugated goat anticat antibody (Santa Cruz, Santa Cruz, CA, USA) for cat IgG and HRP-conjugated goat antihuman IgG for human sera (Santa Cruz, sc2428) was diluted 1:3,000 in PBS-Tween plus 1% (w/v) skimmed milk powder (BioShop Canada, Burlington, ON, Canada). The results of the standardization of the ELISA revealed that the optimal amount of the recombinant S and RBD proteins to use in ELISA ranged from 1 μg to 2 μg/ml in 50 μl buffer, whereas the optimal dilution of SARS-CoV-2-positive cat and human serum ranged from 1:200 to 1:800, and the antispecies secondary antibodies were diluted to 1:3,000 (as per supplier instruction).

The cutoff value was calculated by using the (receiver operating characteristic (ROC) curve analysis to determine the OD value range of positive samples. The ELISA cutoff value was determined by the addition of three standard deviations to the mean OD value of the negative control sera in each plate. OD values above the cutoff value were taken as positive. The cutoff value was recalculated with a ROC curve analysis by using a Med-Calc program (31). Thus, a cutoff OD value of 0.692 (sensitivity: 78.57%; specificity: 93.81%) was determined for the in-house indirect S-ELISA and an OD value of 0.493 (sensitivity: 100%; specificity: 98.23%) for the RBD-ELISA.

### Analysis of Cat Sera for Antibodies to SARS-CoV-2

After standardization of the ELISA, the two groups of cat sera before COVID-19 and during COVID-19 were analyzed for the presence of antibodies to SARS-CoV-2. For this, a 96-well plate (Nunc MaxiSorb, Thermo Fisher Scientific) was coated overnight at 4°C with 100 ng of either S or RBD proteins/per well in 50 μl PBS buffer (Sigma, C-3041). The next morning, plate was washed three times and non-specific interactions blocked for 1 h at room temperature with a blocking buffer (PBS containing 3% (w/v) skim milk powder and 0.1% Tween 20). This was followed by wash steps, a volume of 100 μl of test sera, diluted 1:400 in PBS-Tween plus 1% (w/v) skimmed milk non-fat powder (BioShop Canada), was added and incubated for 2 h at room temperature (20°C). Each serum sample was tested in duplicate, and each test plate included duplicate negative sera as indicated in the standardization of ELISA. Once again, wash step was performed and HRP-conjugated goat anticat antibody (Santa Cruz Biotechnology, Cat. No. sc2428), diluted 1:3,000, was added to the wells and incubated at RT (20°C) for 1 h. One hundred microliters of TMB substrate (Thermo Scientific-C34021) solution was added to each well and incubated at room temperature for 10 min. The reaction was stopped by adding 50 μl/well of 2 M H_2_SO_4_, and absorbance (optical density) was measured at 450 nm using a microplate reader (SLT-Spectra, SLT Lab Instruments, Crailsheim, Germany). The ELISA cutoff value was determined as explained in the standardization of ELISA, thus a cutoff OD value of 0.692 for the in-house indirect S-ELISA and an OD value of 0.493 for the RBD-ELISA.

### Comparison With Commercial ELISA Tests

To assess the reliability of the performance of our in-house indirect S- and RBD-ELISA tests, along with cat sera, sera from human patients were employed which were positive for SARS-CoV-2-specific antibodies by a commercial ELISA (Roche) antibody assay and positive for SARS-CoV-2 RNA by a real-time RT-PCR. Similarly, prechecked negative human sera by ELISA and real-time RT-PCR were used as negative control.

### Surrogate Virus Neutralization Test

In order to detect neutralizing activity against SARS-CoV-2-RBD of the spike protein, a commercial (GenScript Inc., Piscataway, NJ, USA) SARS-CoV-2 Surrogate Virus Neutralization Test Kit was used. This test detects blocking antibodies for binding of SARS-CoV-2-RBD to the ACE2 receptor on a cell surface in an ELISA system. In this study, the cat sera found to be positive in S- and RBD-ELISA were tested by surrogate virus neutralization test, as described by the manufacturer (GenScript Inc., Piscataway, NJ, USA).

### Analysis of Serum Samples for Antibodies to FCoV

Cat sera found to be positive for SARS-CoV-2 antibodies were analyzed for the presence of antibodies to FCoV (Bionote, Anigen, FCoV Antibody and Biogal, FCoV Immunocomb ELISA) according to the manufacturer's instructions.

### Hematological and Biochemical Analyses

Reference values were taken from IDEXX kit instructions used in the laboratory of the Department of Internal Medicine. The reference values for alanine aminotransferase (ALT) and alkaline phosphatase (ALP) were taken from a previous study published by Natalie and others (32). Blood samples from cats were analyzed in detail by a complete blood hemogram-histogram; specifically, total white blood cell counts (WBC), red blood cell counts (RBC), hemoglobin, hematocrit, lymphocyte, neutrophil, monocyte, and platelet counts by using the IDEXX ProCyte Dx™ instrument as described by the manufacturer (IDEXX Procyte Dx Reagent Kit).

Samples from cats were also analyzed for blood biochemistry; ALT, ALP, total protein, blood urea nitrogen (BUN), creatinine levels were measured by the IDEXX Catalyst One instrument as described by the manufacturer (IDEXX Catalyst Chem 10 and IDEXX Catalyst Chem 17).

### Statistical Analysis

Data were analyzed using MedCalc (version 20.010) software. Chi-square test was used to compare the proportion of serological results to age and gender of cats.

## Results

### Assessment of S- and RBD-Specific ELISAs Used in This Study

**Figure 1:**
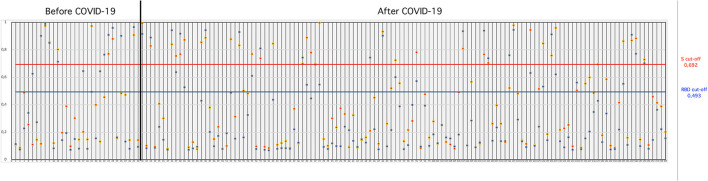
OD values of cat sera collected “before COVID-19” and “during COVID-19” tested in the S- and RBD-ELISAs. The blue horizontal line (bottom line) indicates the “cut-off” value for the RBD-ELISA and the red horizontal line (upper line) indicates the “cut-off” value for the S-ELISA.

SARS-CoV-2-S- and SARS-CoV-2-RBD-specific ELISAs, established in house, were used to detect the presence of antibodies to SARS-CoV-2 in serum collected from cats. The OD cutoff value of the in-house ELISAs were set at an OD of 0.692 for S-ELISA and 0.493 for RBD-ELISA. Of the 155 cat sera analyzed, 26.45% (41/155) tested positive in the in-house S-ELISA, whereas 28.38% (44/155) tested positive in the in-house RBD-ELISA (Figure 1). Thirty-four (21.9%; 34/155) cat sera tested positive in both the S- and RBD-ELISA (Figure 1), five sera were positive only in S-ELISA, and nine sera were positive only in RBD-ELISA (Figure 1). Interestingly, the six sera which were taken before the first reported COVID-19 patient in Istanbul, Turkey, were positive with the RBD-ELISA but negative with the S-ELISA, one was positive by the S-ELISA (Figure 1). All control cat sera were negative for SARS-CoV-2-specific antibodies by both ELISAs.

Both ELISAs were analyzed by kappa statistics, and the kappa value was determined as 0.76. This kappa value indicates that both ELISAs have an excellent agreement based on established criteria reported previously (31).

According to results obtained in the assessment study, a cat serum was defined as positive for SARS-CoV-2-specific antibodies when the serum reacted positive in both, the S- and RBD-ELISAs (agreement in both tests).

### Antibodies to FCoV and SARS-CoV-2 in Cat Sera Collected Before COVID-19

Thirty cat sera obtained before the first COVID-19 patient was reported in Turkey were analyzed for the presence of FCoV-specific antibodies by ELISA. When these 30 cats were tested for antibodies to SARS-CoV-2, six (20%) cats were found to be positive for SARS-CoV-2 by both the S- and RBD-ELISAs (Table 1). OD values of six positive sera ranged between 0.850 and 0.973 in the S-ELISA and 0.710 and 0.987 in the RBD-ELISA (Figure 1). Among the six SARS-CoV-2 seropositive cats, five cats had antibodies to FCoV. One cat had no antibodies to FCoV, and the OD values of this cat to SARS-CoV-2 were 0.801 in the S-ELISA and 0.712 in the RBD-ELISA (Table 1; Figure 1).

**Table 1:** Cut-off values and number of positive and negative cat sera as determined by the S- and RBD-ELISA.

	**Cutoff value** **(optical density)**	**Positive**	**Negative**	**Positive sera taken** **“before COVID-19”**	**Positive sera taken** **“during COVID-19”**
S-ELISA	0.692	41	114	7	34
RBD-ELISA	0.493	44	111	12	32
Positive in both S- and RBD-ELISA		34		6	28
Positive only in S-ELISA		5		1	5
Positive only in RBD-ELISA		9		6	3

*A total 155 cat sera comprising 30 sera “before COVID-19” and 125 sera “during COVID-19” were analyzed*.

### Antibodies to FCoV and SARS-CoV-2 in Cat Sera Collected During COVID-19

One hundred twenty-five cat sera collected during the COVID-19 pandemic were analyzed for the presence of FCoV antibodies. When these 125 cat sera were analyzed for antibodies to SARS-CoV-2, 28 (22.4%) cats were found to be positive for SARS-CoV-2 by both ELISAs (Table 1). OD values of the 28 positive sera ranged between 0.750 and 0.995 for the S-ELISA and 0.670 and 0.944 for the RBD-ELISA (Table 1; Figure 1). Among the 28 SARS-CoV-2 seropositive cats, 22 cats had antibodies to FCoV (Table 2).

**Table 2:** Demographics, clinical, hematological, and biochemical findings in *SARS-CoV-2 seropositive cats*.

**Clinical signs and demographics**						**Circulatory, liver, and renal functions of SARS-CoV-2–positive cats**					
		**SARS-CoV-2 positives** **(***N*** = 34)**		**FCoV positives** **(***N*** = 27)**		**Hematological findings** **and reference values**	**L**	**H**	**Biochemical findings** **and reference values**	**L**	**H**
		**Before COVID-19** **(*n* = 6)**	**During COVID-19** **(*n* = 28)**	**Before COVID-19** **(*n* = 5)**	**During COVID-19** **(*n* = 22)**						
Age											
	0–3	6	15	5	14	HCT:	9 cats:	3 cats:	Creatinin:	3 cats:	6 cats:
	4–7	0	8	0	5	30.3–52.3	18–27	53–56	0.8–2.0	0.2–0.7	2.3–6.1
	8–11	0	5	0	3						
Gender											
	F	5	18	4	16	HGB:	8 cats:	1 cat:	BUN:	2 cats:	2 cats:
	M	1	10	1	6	9.8–16.2	5.8–9.2	17.5	16–36	8–9	42–106
Breed											
	Cross	5	24	4	21	RBC:	2 cats:	0	Total protein:	0	2 cats:
	Pure	1	4	1	1	6.54–12.2	3.5–6.15		5.7–8.9		9.1–9.3
Fever		1	2			WBC: 5.87–17.02	7 cats: 2.8–4.7	2 cats: 19.8–58.5	ALT: 19–71	0	8 cats: 78–253
Depression/dullness		3	2			Neutrophil: 2.3–10.29	1 cat: 0.68	6 cats: 16.1–42.5	ALP: 6–46	0	2 cats: 54–117
Anorexia		2	3			Lymphocytes: 0.92–6.88	11 cats: 0.67–0.69	4 cats: 6.9–12.01			
Weight loss		3	3			Monocytes: 0.05–0.67	0	8 cats: 0.69–0.87			
Respiratory signs (cough, dyspnea, excretion)		2	6			Platelets: 151–600	8 cats: 25–96	2 cats: 660–686			
Gastrointestinal (diarrhea, constipation, vomitus)		2	8								
Urinary (renal insufficiency, cystitis)		1	5								
Neurological		3	2								
Skin lesions		0	6								

*M, male; F, female; H, high; L, low. Reference values were taken from IDEXX kit instructions used in the laboratory of the Department of Internal Medicine. The reference values for ALT and ALP were taken from a previous study published by Natalie and others (32)*.

### Surrogate Virus Neutralization Test

Cat sera (6) positive in S and RBD-ELISA collected before COVID-19 were all negative in surrogate virus neutralization test (sVNT) test. When 28 cat sera positive in S- and RBD-ELISAs collected during COVID-19 tested by sVNT test, three cat sera were found to be positive. Two of these sera were also positive for FCoV antibodies and one cat was living with an owner who recovered from the COVID-19 disease.

### Signalments and Clinical Signs in SARS-CoV-2 Seropositive Cats (*n* = 34)

The signalments and clinical signs of the SARS-CoV-2 seropositive cat cohort at the time of blood collection are shown in Table 2. Most of the seropositive cats (*n* = 21) were below 3 years old. Similarly, 67% (23/34) of the cats were female and the remaining 33% (11/34) were male. Seropositive cats were mainly (85%) cross-breeds (*n* = 29) and only five cats (15%) were pure breeds (Table 2). Seropositive cats exhibited mainly respiratory (*n* = 8), gastrointestinal (*n* = 10), renal (*n* = 6), and skin lesions (*n* = 6). A high body temperature was measured only in three seropositive cats (Table 2).

### Hematological and Biochemical Findings in SARS-CoV-2 Seropositive Cats

Results of the hematology and biochemistry are shown in Table 2. HCT (*n* = 9), HGB (*n* = 8), WBC (*n* = 7), and platelets (*n* = 8) were low in several seropositive cats. Lymphopenia was seen in 11 cats (33%), and these lymphopenic cats had high titers of antibodies to the S and RBD proteins. The number of neutrophils and monocytes were above the normal in six and eight cats, respectively (Table 2). Creatinine was found to be low in three cats and high in six cats. The liver enzyme ALT level was high in eight cats (Table 2), and these cats also had a very high titer of antibodies to the S and RBD proteins.

### Statistical Analysis

The seroprevalence of age and gender was not statistically significantly different between seropositive and negative cats (*p* > 0.05).

## Discussion

A considerable number of reports indicated that domestic and wild felids have been shown to be highly susceptible to natural and experimental SARS-CoV-2 infections (9, 11, 12, 17), indicating that cats can be infected with both, FCoV and SARS-CoV-2; therefore, serological cross-reaction might complicate serological diagnosis. So far, no comprehensive data are available on the analysis of cat sera for the presence of antibodies to SARS-CoV-2 before the emergence of SARS-CoV-2, December 2019 (33), and there is no report on the seropositivity of cats to SARS-CoV-2 in Turkey. Therefore, in the present study, the presence of SARS-CoV-2-specific antibodies within the cat population in Istanbul, Turkey was investigated.

SARS-CoV-2 seems to have originated from bats (1, 17) although this has not been proven; also, the involvement of an intermediate mammalian host such as the pangolin or raccoon dogs (8, 10, 34) before transmission to humans has been discussed. COVID-19 patients can transmit SARS-CoV-2 to domestic and large cats while cat to human transmission has not been reported yet (12, 14, 17). Seroprevalence studies in cats indicated that cats can be infected with SARS-CoV-2 presumably by their SARS-CoV-2-infected owners, showing mostly mild disease (17). Natural SARS-CoV-2 in cats has been reported in several countries, e.g., in Belgium (25), Hong Kong (35), USA (36), France (20), Spain (23, 24, 26), Germany (13), UK (17), Italy (37), Switzerland (38), China (14), and SARS-CoV-2-positive cats have been reported to the OIE from Russia, Denmark, Sweden, Chile, Japan, Brazil, and Argentina (39).

In Wuhan, China, 102 cats were analyzed for the presence of antibodies to SARS-CoV-2 by ELISA, and 15 of 102 (14.7%) cat sera were found to be SARS-CoV-2 positive (14). Eleven of the 15 ELISA-positive cats had neutralizing antibodies with titers ranging from 1/20 to 1/1,080, which is rather high compared with titers reported in other cat studies. No obvious clinical signs were observed in SARS-CoV-2 seropositive cats. Three of the SARS-CoV-2-positive cats were living in households with COVID-19 patients, indicating that the close contact to the SARS-CoV-2-positive owners could be the most likely source of infection (“reverse zoonosis”). Ten out of 39 serum samples collected before the emergence of SARS-CoV-2 in December 2019 were positive for SARS-CoV-2 antibodies (14). The authors indicated that no serological cross-reactivity was observed in their serological assay between antibodies against SARS-CoV-2 and type I or II feline coronavirus (14). In Germany, a total of 920 cat sera were screened by an indirect multispecies ELISA, indirect immunofluorescence test (iIFT), and neutralization test specific for SARS-CoV-2 antibodies. Overall, six (0.69% or 6/920) serum samples tested positive for antibodies to SARS-CoV-2 by both ELISA and iIFT, and two of these sera were also positive in the virus neutralization assay (13). The prevalence of SARS-CoV-2 antibodies in cats in Germany is low when compared with other countries. This could be due to the fact that the German serum samples were collected during a time period (March–April 2020) when the incidence of SARS-CoV-2 infections in the German population was low. In contrast, in France, a rather high seroprevalence of SARS-CoV-2 antibodies was found in cats, ranging from 21 to 53% (21). In another study performed in France, 22 cats that had contact to SARS-CoV-2-infected owners were tested for the presence of SARS-CoV-2 RNA by RT-PCR, and sera from 16 cats obtained before the emergence of COVID-19 were analyzed for the presence of antibodies by ELISA. SARS-CoV-2 RNA was detected in one cat, and this cat also tested positive for carrying antibodies to SARS-CoV-2 by ELISA. No antibodies to SARS-CoV-2 were detected in cats sampled before the emergence of COVID-19 (20). In Texas, USA, eight of 17 cats living in COVID-19-affected households were found to be positive either for SARS-CoV-2 RNA or SARS-CoV-2-specific neutralizing antibodies (17, 19). In a study performed in Italy, 5.8% of cats had neutralizing antibodies to SARS-CoV-2 (17, 22). The authors indicated that 22 cat sera originated from COVID-19-affected households (17, 22). In a study performed in Spain, 114 stray cats were tested for SARS-CoV-2 and four cats (3.51%) were found to be positive for SARS-CoV-2 antibodies (24). In another study in Spain, a cat living with a family with several members affected by COVID-19 was analyzed and found to be positive for SARS-CoV-2 RNA. The cat and also another cat in the same household seroconverted against SARS-CoV-2 (23). Similarly, a cat in Belgium diagnosed with severe acute respiratory syndrome, and living with a COVID-19-positive owner, tested positive for SARS-CoV-2 RNA and developed a SARS-CoV-2-specific antibody titer of 1:512 (25). In a study performed in Croatia, 131 cats were tested and 10 cats (0.76%, 10/131) were found to be positive for SARS-CoV-2 antibodies (40).

In the present study, evidence of the presence of antibodies to SARS-CoV-2-specific S and RBD proteins was found in cats sampled before COVID-19 and during COVID-19. A total of 155 cat sera were tested, with 34 (21.9%) being positive for both, the S and RBD proteins. The owner of one seropositive cat had recovered from COVID-19 about 1 month before the cat was sampled, and this cat had high antibody titers to both, S and RBD proteins. Results of our study indicate that SARS-CoV-2 is circulating in cat populations in Istanbul, Turkey, and that SARS-CoV-2 infections in cats were most likely acquired from SARS-CoV-2-positive cat owners. Unfortunately, only limited data on the health status of the cat owners is available since they were not willing to provide detailed information about their SARS-CoV-2 infection status. Importantly, all cats analyzed in this study were household cats and had no access to the outside A similar transmission of SARS-CoV-2 from COVID-19 patients to cats has been shown in numerous cases previously (17, 22, 23). Interestingly, six of the 34 SARS-CoV-2 seropositive cats were sampled before the emergence of COVID-19 in humans, i.e., before December 2019. This could be due to: (i) a cross-reactivity of immune responses between FCoV and SARS-CoV-2, most likely to the nucleocapsid protein; or (ii) a SARS-CoV-2-like virus circulating in cats in Istanbul. Since we do not have any virus/RNA isolates from these cats, the latter possibility is rather speculative. It is most likely that SARS-CoV-2 seropositivity in cats sampled before the COVID-19 is due to cross-reacting antigens between SARS-CoV-2 and FCoV, since five of these six SARS-CoV-2 seropositive cats also had antibodies to FCoV. Only one cat was negative for FCoV antibodies. This observation warrants further investigations.

In the present study, two ELISA tests, one for the SARS-CoV-2 spike protein and one for the RBD region of the spike protein were used to determine the antibody status of cats. The kappa value (0.76) obtained indicates that both the S- and RBD-specific ELISAs had an excellent agreement based on established criteria reported previously (31). This shows that either one of these tests can be used for prescreening cats for antibodies against SARS-CoV-2 in cats. Since we do not have BSL-3 facilities at our institution, the ELISA-positive sera could not be tested by a classical virus neutralization assay. Therefore, we tested the ELISA-positive cat sera for the presence of neutralizing antibodies using the GenScript ACE2-based SARS-CoV-2 surrogate virus neutralization test (sVNT); three of our ELISA-positive cat sera were found to be sVNT positive, indicating that these cats were exposed to SARS-CoV-2. It is important to note that previous cat studies revealed that some ELISA-positive sera were negative in classical virus neutralization assays (13, 14, 21). The respective authors attributed the negative virus neutralization results in the delayed production of neutralizing antibodies (13). In addition, results of experimental SARS-CoV-2 infection of ferrets indicated that SARS-CoV-2 neutralizing antibodies in the ferret COVID-19 model can only be detected about 2 weeks after the initial detection of SARS-CoV-2-specific antibodies using an iIFT (13, 40). A similar situation as mentioned above could be the reason that only three out of 34 SARS-CoV-2-positive sera tested also positive in the surrogate sVNT assay. Therefore, based on the results obtained by others, the SARS-CoV-2 ELISA-positive but sVNT-negative cats identified in this study were either in early stages of SARS-CoV-2 infection or exposed to a feline coronavirus inducing cross-reactive antibodies to the SARS-CoV-2-S and SARS-CoV-2-RBD proteins. The latter one most likely applies to the six ELISA-positive cats sampled before the emergence of COVID-19, since five of these cats also had antibodies to FCoV. Future studies are needed to investigate if there is cross-reactivity between SARS-CoV-2 and FCoV, although several reports indicate no cross-reactivity (10, 13, 21, 41).

Mild clinical signs or no signs were reported in cats infected with SARS-CoV-2 (10, 20, 35). In the present study, the majority of the seropositive cats (*N* = 21) were between 0 and 3 years old, and respiratory (8/34) and gastrointestinal (10/34) signs were prominent with low hematocrit (9/34), hemoglobin (8/34), lymphocyte (11/34), and platelet (8/34) levels. Alanine aminotransferase levels were increased in eight seropositive cats. These findings are similar to those seen in another cat study (32) and people infected with SARS-CoV-2 (42).

In conclusion, this is the first report describing antibodies to SARS-CoV-2 in cats in Istanbul indicating the risk of cats getting infected with SARS-CoV-2. It is important that cat owners are aware that when they acquire COVID-19, they should apply preventive measures to avoid transmission of the virus to their cats (or other susceptible pets). For this reason, suspected companion animals should be monitored for SARS-CoV-2 infection and separated from naive people and susceptible animals until recovery. Currently, the CDC (43) and OIE (44) recommend the separation of people infected with COVID-19 from their companion animals. Also, the European Advisory Board on Cat Diseases (ABCD) (45) advises isolating positive animals from unexposed ones. For preventive measurements, a “*One Health*” approach should be implemented, with the inclusion of Public Health and Veterinary Services.

## Data Availability Statement

The raw data supporting the conclusions of this article will be made available by the authors, without undue reservation.

## Ethics Statement

Ethical review and approval was not required for the animal study because as indicated in the study, only the sera taken from the sick animals that submitted to the Veterinary Faculty of Istanbul University for the diagnosis were investigated. Informed consent was taken from the cat owners. National and international guidelines were followed for the care of animals during the study. Written informed consent was obtained from the owners for the participation of their animals in this study.

## Author Contributions

AY, HY, BK, NT, JAR, and MI: conceptualization. HY, GR-S, J-RS, PC, JAR, and MI: supervision. AY, HT, AK, OI, AB, and EO: data collection. AK, OI, AB, EO, HT, and RK: sample collection. AY, HY, AK, NT, EO, BK, RK, J-RS, DB, GR-S, DL, PC, MI, and JAR: methodology. AY, HY, DB, GR-S, J-RS, DL, PC, MI, and JAR: recombinant protein production. AY, OI, AB, HT, DB, GR-S, J-RS, DL, and PC: laboratory analyses and validation. AY, HY, MI, and JAR: writing the original draft. AY, HY, NT, AK, BK, RK, DL, PC, MI, and JAR: review and editing. All authors have read, discussed the results, and agreed to the final version of the manuscript for publication.

## Funding

This study was funded by the Istanbul University-Cerrahpasa (BAP Project No: TSG-2020-34882). The study was partially supported by the NIAID Centers of Excellence for Influenza Research and Surveillance under contract number HHSN 272201400006C, the MCB Core of the National Institute of General Medical Sciences (NIGMS) of the National Institutes of Health under award number P20GM130448, and the Department of Homeland Security Center of Excellence for Emerging and Zoonotic Animal Diseases under Grant Number HSHQDC 16-A-B0006 to JAR. The funding from UK Research and Innovation (UKRI) Biotechnology and Biological Sciences Research Council (BBSRC) Grants (BBS/E/I/00007031 and BB/S013792/1) also contributed to this study.

## Conflict of Interest

The authors declare that the research was conducted in the absence of any commercial or financial relationships that could be construed as a potential conflict of interest.

## Publisher's Note

All claims expressed in this article are solely those of the authors and do not necessarily represent those of their affiliated organizations, or those of the publisher, the editors and the reviewers. Any product that may be evaluated in this article, or claim that may be made by its manufacturer, is not guaranteed or endorsed by the publisher.
